# The good executive in the wake of COVID-19: using story completion to study the rise and development of alternative selves

**DOI:** 10.3389/fpsyg.2025.1499311

**Published:** 2025-06-09

**Authors:** Claudia Manca, Marcello Russo, Ludovica Leone, Richa Gaavar

**Affiliations:** ^1^Department of Management, University of Bologna, Bologna, Italy; ^2^Department of Economics “Marco Biagi”, University of Modena and Reggio Emilia, Modena, Italy

**Keywords:** alternative self, cultural schemas, executive, story completion, work devotion

## Abstract

**Introduction:**

The COVID-19 pandemic was a turning point that had the potential to shape how individuals manage their work-family interface, possibly bringing the emergence of alternative self-definitions that are tied to changing cultural norms. This study aims to explore whether and how executive managers have developed an alternative self after the pandemic, shedding light on the interplay between cultural norms, gendered assumptions, and executive identity formation.

**Methods:**

To understand the development of self-concept as intertwined with cultural norms and gendered assumptions, we used the story completion method that requires participants to create autonomous stories based on a provided “cue.” We analyzed stories written by 32 executives based in Italy and the United Kingdom (14 males and 18 females) who reflected upon contemporary discourses on what it takes to be a good executive when the scaffolding between work and family life has been disrupted.

**Findings:**

This study highlights that the pandemic has served as a turning point that elicited alternative views about self and cultural meanings for the executives. These alternative views trigger three distinct patterns for the development of alternative selves i.e., *becoming a work-life balance advocate*, *becoming a family man*, and *reasserting the ideal worker*.

**Discussion:**

This study sheds new light on the dynamics at the intersection between individual experiences in work and family management and broader societal expectations and cultural norms. It highlights how executives adapt their self-concept in response to major turning points, and how cultural norms conflate into this process. This allows us to offer new insights into the relationship between turning points, identity shifts, and societal expectations.

## 1 Introduction

The way people manage their work-family interface has changed over time due to key turning points in our society. Turning points are events that generate significant alterations in the meaning, purpose, or direction of a person’s life (Pillemer, 2001; Wethington et al., 1997). These events can be triggered by external shocks such as wars, migrations, and economic downturns that affect how individuals manage their lives and may elicit alternative views about the perception of self and cultural meanings (Dumas and Sanchez-Burks, 2015). Yet, beyond an immediate disruption, there remains limited understanding of whether and how major turning points can trigger a more profound and enduring shift in attitudes, behaviors, and identities related to the management of the work-family interface.

In this respect, the COVID-19 pandemic may have constituted a significant turning point for many. In particular, the abrupt and widespread shift to remote arrangements significantly altered the spatial and temporal boundaries of work, prompting people to reinterpret and renegotiate their roles, responsibilities, and priorities across multiple domains (Choudhury et al., 2021; Kniffin et al., 2021). In this paper, we aim to explore these alterations for professionals, specifically focusing on executive managers (hereinafter executives), who faced a sudden overlap between their personal and professional lives due to COVID-19. This overlap compelled them to confront previously compartmentalized roles, potentially eliciting alternative views of the self as intertwined with changing cultural norms.

This line of inquiry is particularly compelling in masculine work cultures, such as those in Italy and the United Kingdom, where rigid norms of overwork and inflexibility historically persist. Indeed, in such contexts, individuals are often expected to orient their lives either toward career or family, typically along gendered lines (cf. Cannito and Scavarda, 2020; Chung, 2023; Chung and Van der Horst, 2020). These cultural expectations reflect two competing cultural schemas: work devotion and family devotion (Blair-Loy, 2003). Work devotion entails an intense commitment to paid labor, epitomized by the “ideal worker” who prioritizes paid work above all else (Acker, 1990). Family devotion, conversely, demands unwavering commitment to caregiving duties, especially from women (Blair-Loy, 2003; de Laat, 2025). Historically, these schemas have drawn strict boundaries between work and family, casting them as mutually exclusive domains. However, the COVID-19 pandemic may have unsettled these boundaries, creating openings to challenge cultural norms and enabling individuals to renegotiate roles, responsibilities, and priorities across domains (de Laat, 2025; Turner et al., 2022). This major shift could have been particularly consequential on executives, whose prevailing occupational script focuses on professional commitments. The archetype of the “good executive” indeed portrays either a man fully focused on the job with a stay-at-home wife, or a woman (preferably single or childless) equally absorbed in her career (Blair-Loy and Amy, 2004; Dumas and Sanchez-Burks, 2015).

Therefore, we investigate whether and how executives, especially in masculine work cultures, have developed alternative self-definitions following the alteration of their work-family routine caused by the COVID-19 pandemic, highlighting the role cultural norms might have played in this process. In order to do this, we embrace the methodological approach of story completion (Clarke et al., 2019; Gravett, 2019) and draw upon the stories shared by 32 executives working in Italy and the United Kingdom. This methodology requires participants not to share their actual experiences but to imagine how a proposed hypothetical scenario would play out based on some initial information provided (Clarke et al., 2017). Unlike interviews, story completion enables us to look beyond the glare of actuality, highlighting the self-redefining role of the alternative experienced by executives during the pandemic. Drawing upon executives’ stories, we outline how self-development and cultural meanings play out when managing the work-family interface. By doing so, we address the call launched by Williams et al. (2016) to integrate research on individual experiences of work and family with an examination of how cultural norms shape attitudes and behaviors at the intersection of these domains. More specifically, we enhance our understanding of how macro-level disruptions, like the recent pandemic, may open space for shifts in cultural norms and self-concept definitions, particularly for individuals belonging to occupational groups in which cultural schemas have historically proven resistant to change (cf. Blair-Loy and Amy, 2004; Dumas and Sanchez-Burks, 2015).

The contribution of the study is multifaceted. First, by drawing on the collected stories, we found that the pandemic brought executives to embrace alternative views around cultural meanings and self-concepts. The abrupt change in the management of the work-family interface made extremely salient in our respondents’ minds an alternative pathway to work devotion, which triggered, at an individual level, the formation and development of an alternative self: a self-redefining counterfactual of who a person might have become if something had turned out differently in the past (Obodaru, 2012). The temporary actualization of an alternative self brought executives to experience some tensions that stem from the perceived incompatibility between alternative self-representations and the embodied image of the good executive as an ideal worker, suggesting that competing schemas still have a firm grasp on our respondents. This allows us to contribute to the ongoing debate of whether cultural schemas hold even while facing major turning points (cf. de Laat, 2025; Cannito and Scavarda, 2020; Chung, 2023). Nonetheless, we also observed that several executives in our sample solved that tension by crafting new occupational scripts, incorporating a new role as wellbeing advocates into their self-concept. This is important as executives not only face distinct pressures and expectations, but their attitudes and approaches to work-life balance can shape organizational norms, influence policy uptake, and collaborators’ work-family decisions, and reflect deeper social and gender dynamics (Adamson et al., 2021).

By integrating research on turning points (Pillemer, 2001; Wethington et al., 1997), alternative self (Obodaru, 2012), and cultural schemas (Blair-Loy, 2001; Williams et al., 2016), we advance our understanding of how macro-level disruptions can catalyze identity renegotiation among professionals and how the work-life interface is managed. We found that the COVID-19 pandemic has been a significant turning point, activating alternative self-concepts that challenged dominant cultural schemas of work and family devotion. While previous research emphasized the resilience of these schemas, our findings reveal that extraordinary contextual shocks can temporarily disrupt their normative power, creating spaces for executives to explore alternative pathways. Nevertheless, the enduring emotional and cognitive pull of the ideal worker norm suggests that identity redefinition remains contested, especially within masculine work cultures. In doing so, we offer an additional perspective of identity negotiation for executives, expanding theoretical conversations about the interplay between societal shocks, cultural meaning systems, and self-concept development at the intersection of work and family life.

## 2 Literature background

### 2.1 The rise and development of an alternative self for executives

The self-concept is a dynamic cognitive structure reflecting multiple self-representations (Markus and Wurf, 1987). Self-representations are images of who the person is in the present—the current self—according to one’s multiple societal roles and group memberships or self-definitions (Higgins, 1987). Self-definitions also include hypothetical self-images, such as the past self (Albert, 1977), the future self (Markus and Nurius, 1986), the relational self (Morandin et al., 2021), the ideal self, and the ought self (Higgins, 1987), which all have a self-redefining power, as they influence how individuals see themselves, set goals, and actively adapt and grow in response to changing circumstances.

On the same line, Obodaru (2012) introduced an additional type of self-image: the alternative self. The alternative self entails representations of what a person might have become in the present if something in the past had turned out differently. Alternative self-representations are often triggered by counterfactual thoughts resulting from exceptional events. These thoughts can give rise to better or worse alternative realities, leading to a potential redefinition of one’s self-concept; in this case, the counterfactual thought is self-redefining and constitutes an alternative self (Obodaru, 2012).

Extraordinary and unusual events can represent turning points that cause significant deviations from norms and routines, making an alternative reality more salient in one’s mind (Habermas and Bluck, 2000; Kahneman and Miller, 1986; Thorne et al., 2004). In this respect, we contend that COVID-19 represented a significant turning point for executives, who are often required to adhere to the cultural norms of the ideal worker, available to work 24/7 and motivated to put work at the first place in life, even when it does not correspond to personal preferences and needs (Blair-Loy, 2003). Depicting the “good executive” as an ideal worker has made it difficult—and even penalizing (Reid, 2015)—for executives to participate in the day-to-day life and activities of their families. By limiting executives’ travel and requiring them to stay home for extended periods, COVID-19 made an alternative self more salient in the executives’ minds. When an alternative self is perceived as better or worse than the current self, it can trigger either positive or negative emotions and attitudes, resulting in executives reassessing their current state (Obodaru, 2012). Hence, we can expect that a perceived discrepancy between the alternative and current self may enhance the motivation for executives to change, shaping important work and family life decisions.

### 2.2 The importance of cultural schemas for the development of the alternative self

The alternative self does not develop in a vacuum, but in a socio-cultural context that influences its development. To describe how cultural meanings conflate with personal experiences to develop the alternative self, we draw on the concept of cultural schema, which is “*an ordered, socially constructed, and taken-for-granted framework for understanding and evaluating self and society, for thinking and for acting*” (Blair-Loy, 2001; p. 689). Cultural schemas enable individuals to identify the aspects they must care about, outlining a clear direction on how to do that, influencing their social and personal identities (Blair-Loy, 2005). Executives’ choices are sculpted by the two cultural schemas based on gendered roles: work and family devotion (Blair-Loy, 2001). As above mentioned, the work devotion schema reflects the deeply rooted cultural assumption of work “*as a potent site of moral prescriptions*” (Williams et al., 2013; p. 211) that demands and deserves continuous time and commitment (Blair-Loy, 2005). Individuals adhering to this schema prioritize work before personal and family needs (Williams et al., 2013). They internalize the ideal worker norm (Acker, 1990), regarding work devotion as an end, worthy of being pursued with utmost passionate devotion (Weber, 1946). Differently, the family devotion schema signals home and family as a woman’s primary commitment (Blair-Loy, 2001). The schema also stipulates the model of ideal motherhood that aligns with Hays’ (1996) *ideology of intensive motherhood*: a cultural structure that posits motherhood as a woman’s sacred duty that is “*child-centred, expert-guided, emotionally absorbing, labour-intensive, and financially expensive*” (p. 129). Thus, a woman is expected to be devoted first and foremost to her family, even if she is working outside the home, and must find contentment, meaning, and intimacy in her role as a mother (Blair-Loy and Erin, 2017).

Williams et al. (2016) identified four identities shaped by cultural schemas and ideal worker norms: the good worker, the good man, the good woman, and the good person. The good worker sees work as a moral duty, aligning with the work devotion schema and the ideal worker representation (Williams et al., 2016). The good man views work as a masculinity contest with unquestioned availability to work as a heroic endeavor (cf. Ely and Meyerson, 2000; Epstein et al., 2014). Conversely, the good woman perceives work as a moral hazard, bearing the burden of conflicting ideal worker and ideal mother expectations (Benard and Shelley, 2010). Finally, the good person regards dedication to work as a class act, with hard work and competence symbolizing strong moral character and purity (Lamont, 1992). Research suggests that social norms intersect with individual identities in influencing the management of the work-family interface.

Each schema demands unwavering commitment, portraying work and family as opposing and mutually exclusive aspects of life (Halrynjo and Lyng, 2009). These schemas have been repeatedly contested owing to women’s increasing participation in the workforce (Blair-Loy, 2001). Recent studies have also suggested that COVID-19 might have accelerated a cultural change, inducing fewer penalties for workers not adhering to these schemas as they increasingly use, for instance, flexible work arrangements for family purposes (cf. Chung, 2023). However, scholars have also argued that these schemas retain a firm cognitive, emotional, and normative influence on individuals (e.g., Padavic et al., 2019). Thus, while the experience of an unprecedented pandemic may have suspended social norms (for further discussions on the topic, see Chung, 2023; de Laat, 2025) and unsettled certain expectations, the durability of cultural schemas suggests that any shifts in self-concept would be fraught with tension and negotiation.

Consequently, in this study, we question whether the alternative selves activated during the pandemic experience, following the change in their work-life routines, led executives to renegotiate their work and family identities and whether these shifts became integrated into their self-concept, highlighting the role that socio-contextual forces might have played in this process. In doing so, we contribute to the ongoing conversation about whether macro-level disruptions like COVID-19 merely pause or shift long-standing cultural norms at the work-family interface (Cannito and Scavarda, 2020; Blair-Loy and Amy, 2004; Dumas and Sanchez-Burks, 2015).

## 3 Research methodology

As we dig into cultural meanings and alternative self-definitions that might deviate from established roles and identities, we decided to use story completion that provides the opportunity to foreground the role of cultural meanings in shaping alternative self-definitions. Indeed, story completion has been presented as a valuable tool not only for accessing psychological meanings but also for exploring social discourses (Clarke et al., 2019). Bruner (1990) considered stories as manifestations of “*culturally-shaped notions in terms of which people organize their views of themselves, of others, and of the world in which they live*” (p. 137). Story completion is a qualitative methodology that was originally employed within the psychoanalytic (clinical) tradition as a projective technique, which involves conducting tests by presenting the subject with an ambiguous stimulus, thereby allowing them to project their hidden emotions and motivations onto the test (Pong et al., 2024). The main aim was to reveal unconscious “truths” by avoiding two barriers: the barrier of awareness, where the subject may not be consciously aware of certain feelings or motivations, and the barrier of admissibility, where the subject might be unwilling to admit certain emotions when directly questioned.

More traditional qualitative research methodologies typically rely on self-report techniques for data collection, such as interviews or focus groups, to provide insights into individuals’ personal experiences and perspectives through their own accounts. Differently, story completion requires participants to craft a story based on an initial stem or “cue” provided to them (Clarke et al., 2019). Notably, story completion does not involve descriptions of events as they occurred but allows researchers to gain insight into the cultural discourses and meanings that enable participants to make sense of the research topic (Clarke et al., 2017). Through stories, we can understand how people produce and re-produce a shared sense of meaning that informs and constrains their identity, actions, and interpretation of reality (Weick et al., 2005).

In accordance with recommendations on constructing story stems (Braun et al., 2019) and research that suggests differences in strategies employed by male and female executives in managing their work-family interface (Reid, 2015), we presented participants with a story stem that featured a variation in the gender of the main character, portraying an executive adhering to the work devotion schema but forced to remain at home due to the lockdown measures in response to the COVID-19 spread.

The story stem was initially piloted with a small group of five managers to understand the potential and pitfalls of the research instrument. Once we assessed the final version of the script, we recruited a convenience sample (Etikan et al., 2016) of 16 executives who were more easily accessible through the researchers’ personal and professional networks. In identifying these executives, we looked for diversity of profiles in terms of gender, provenance, and industry. Starting with these participants, we asked them to refer others in the same company who fit the study criteria (i.e., holding an executive role position and having stayed at home for most of the pandemic period due to social restrictions). This combination of convenience and snowball sampling was employed as the participants were business executives, a group that is often difficult to access when considering topics that pertain to the management of the work-family interface. We reached a final number of 32 business executives, employed in telecommunication, consumer-goods, machinery, and educational industry. Participation was voluntary, and confidentiality and anonymity were ensured throughout the study, with all data handled in compliance with applicable data protection regulations. Participants were also informed that there were no potential risks of participating in the study and were encouraged to get in touch with the researchers if they had any questions or concerns. The final sample included 14 male and 18 female executives who were randomly assigned to a specific stem reflecting one male or female executive forced to remain at home due to the pandemic. 62% of the respondents had at least one child. The average age was 48 years, with an average tenure in a managerial role of 16 years.

The final stems were the following:

- Male executive.
  Marc is always busy due to his role as Executive Director. He is firmly convinced that a good manager should be available to work 24/7 so this situation is all right for him also considering that his partner does not have a job and takes care of the family. Since February 2020, due to COVID-19 pandemic, Marc has spent almost all days at home with his partner and children, participating as never before in the day-to-day life of his young family.
- Female executive.
  Rebecca is always busy due to her role as Executive Director. She is firmly convinced that a good manager should be available to work 24/7 so this situation is all right for her also considering that her partner does not have a job and takes care of the family. Since February 2020, due to COVID-19 pandemic, Rebecca has spent almost all days at home with her partner and children, participating as never before in the day-to-day life of her young family.

Participants were invited to read the story stem and then write what would happen next. They were informed that there were no right or wrong ways to complete the story and were required to invest at least 10 min in writing. We asked participants to complete the story based on the assigned stem, to reflect not on real-life events, but on contemporary discourses upon which they try to make sense of their own individual experiences (Kitzinger and Powell, 1995). We asked them to do so in a way to reflect such experiences in their stories, along with their own attitudes and beliefs. This instruction made it possible for us to use the language of the “self” although participants told their stories in third person to favor candor.

In developing the analysis, we built upon a Burkean approach to stories (Burke, 1969) by considering storylines that are fragmented and non-linear, as well as coherent narratives with a clear beginning, middle, and an end. To analyze the data, we adapted the three-level framework to narrative research from Bamberg (1997). This allows us to entail three complexity aspects: the (fictional) evolutionary timeline of the alternative self-development, the role of the dominant cultural schemas, and the positionality of the main character. Subsequently, we implemented the three levels of data analysis described below.

**Level 1: storylines**. First, we created complete storylines by connecting story elements that arose from the individual accounts into coherent plots. These elements include: (1) the *exposition*, an opening act that reveals the emergence of a conflict or a precipitating event—this element corresponds to the “stem” provided to respondents; (2) the *climax*, representing the turning point of suspense; (3) the falling actions that follow the climax to let the protagonist deal with the situation; (4) the *resolution*, or end of the story, in which the conflict is solved. Most of the stories encompassed all these elements. During coding, we primarily focused on the management of work-family interface.

**Level 2: context**. To acknowledge the role of the broader sociocultural context, we coded for the elements connected with cultural schemas. Drawing on the good worker, good man, good woman, and good person ideals (Williams et al., 2016), we contend that cultural schemas could be found in the gendered assumptions of (a) *work as a masculine contest*, (b) *work as a moral act*, (c) *work as a moral hazard*, and (d) *work as a class act*. Based on these contextual elements in the storyline, we could see how the main characters developed awareness on these assumptions and either rejected or reasserted them.

**Level 3: positionality**. Finally, we analyzed how the storylines were negotiated by different executives based on their social positioning—their gender, the gender of the main characters of their stories, and how these have been represented throughout the narrative (e.g., workaholic, dad, married, ambitious professional, etc.).

The analysis was an iterative process between these three levels, driven by the constant comparison of differences and similarities between the stories that allowed us to identify three emerging patterns presented in the findings. Although story completion is inherently subjective as it draws on interviewers’ interpretations of provided accounts, the research team took several steps to establish the validity and reliability of the method to ensure the trustworthiness of the findings. The authors ensured validity by providing participants with a clear and relevant story stem to elicit meaningful responses. The final story stem was developed after obtaining feedback from colleagues who are familiar with qualitative research and after a pilot test. Furthermore, the authors ensured that the story stem and instructions were consistent for all the participants to minimize variability. The data analysis process involved two authors independently coding the data as soon as they started receiving stories. Having multiple coders analyzing the stories using the same framework helped to keep individual authors’ biases in check, ensuring that they did not unduly influence the data analysis and produce reliable results. Throughout the data analysis process, the authors conducted regular meetings to discuss the emerging themes and to ensure that data analysis was closely aligned with the participants’ stories.

## 4 Findings

We found that the COVID-19 pandemic has made salient in the respondents’ minds an alternative reality. This alternative pathway triggered tensions between past self-definitions and alternative selves, arising at the level of self-representations linked to cultural schemas. The different ways in which the narrators worked through these tensions outline three different storylines corresponding to alternative decisions pertaining the management of the work-family interface: (i) *reframing opposing self-definitions as synergic: becoming a work-life balance advocate*, (ii) *withdrawing from the past self: becoming a family man*, and (iii) *withdrawing from the alternative self: reasserting the ideal worker*.

### 4.1 Storyline 1. Reframing opposing self-definitions as synergic: becoming a work-life balance advocate

The first story portrays the transformation of the good executive from a tireless workaholic to a work-life balance advocate. This storyline, told by most respondents (25 out of 32), begins with the executive experiencing the removal of those structures supporting the separation between work and family roles, including separate spaces, work schedules, school times for kids, and idle times in and around work. This makes it difficult for them to simultaneously meet expectations from both domains, triggering a contraposition between the self-representation of the good executive as an ideal worker and the actualized alternative counterfactual of the involved parent.

*“So, Marc was stressed enough staying at home most of the time, trying to keep his “business as usual” which was obviously very difficult as other “residents” of the house were trying to get along with their lives (at first, kids in the background of the Zoom calls were the most innocent incident happened to our Executive Director)” (story #4, male respondent).*

*“[Story on Rebecca] They have seen that there are tensions in the house due to work, and in reverse, there are tensions in the home life because of the closeness that they are all on top of each other each day” (story #7, male respondent).*

To deal with this contraposition, Marc and Rebecca start negotiate meeting rules and availability expectations with both family and colleagues, forcing the introduction of some family time in their daily schedules. They increasingly reveal family commitments to collaborators, and delegate more responsibilities to their staff.

*“Of course, Marc's wife would be at the kids’ back and call, but by purely being at home, he started to understand that his family wanted to spend more time with him. They quickly got into a new routine, where they would sit down at the breakfast table every morning and have breakfast together as a family” (story #25, female respondent).*

These activities progressively reshape Marc and Rebecca’s assumptions about work and family as conflicting domains, rejecting work as a masculine contest. Through their narratives, the executives reflect upon how supporting a good work-life balance over inflexible schedules can help people be more fulfilled in life and, thereby, more productive and motivated at work. They also consider valuable household responsibilities and activities revolving around care, highlighting how these develop skills that are also crucial in the work context.

*“Although initially this reflection quickly disappeared from Rebecca's thoughts, as she was busy solving much more important problems, it gradually became almost a fixed thought. Maybe it was worth experimenting with some new ways of managing your collaborators? Perhaps there are also other places, situations, relationships that allow you to develop managerial skills outside the workplace?” (story #11, male respondent).*

Narrators also reflect on the danger of framing work as a moral hazard for women. In one of the stories, the good mother’s imperative to solely focus on childcare responsibilities left Marc’ spouse unfulfilled in life, reflecting negatively also on Marc’s satisfaction with life in general.

*“But, on the other side, Marc uncovered the fact that his wonderful wife, who put her career on pause to take care of the family, was slowly losing interest in anything beyond the daily routine of kids, school meetings and cooking. That was a very alarming discovery because Marc knew that it was a sign of Mary losing interest in herself, her life, dreams, and aspirations” (story #4, male respondent).*

In another story, Rebecca found inspiration in a colleague, Elaine, who became the living example of how real success requires women to avoid either/or choices and find ways to combine work commitment with parental duties to “have it all”:

*“Rebecca appreciates how supportive Elaine has been throughout the transition and for being a role model of 'professional working woman'. She understands that between an executive leader, a partner and a mom, there's a way to have it all” (story #26, female respondent).*

One narrator challenges the assumption of work as a moral act through Marc, suggesting that executive identities have long been framed around work devotion to justify years spent overlooking their families to fulfill societal and organizational expectations.

*“The ED [Executive Director] realized that by making himself available 24/7 to the business, he was cheating his family of his time and love. The ED took a step back and acknowledged that he could go better than just provide the money for the family” (story #21, female respondent).*

In all these stories, this problematization of the cultural meanings underlying work devotion led the protagonists to rethink themselves as good executives, who are now conceived and depicted as professionals capable of balancing work and family life, promoting sustainable ways of working. These new representations were developed through several narrative elements, such as kids’ aspirations, societal concerns for sustainability, and common ways of doing things, outlining new managerial paradigms.

*“Marc also feels that a more balanced approach to work and life, with less commuting, less time outside of the family, less travel, less junk food, can contribute to a greener and more sustainable world with less traffic, less pollution, and less gentrification of cities, and that resonates with his kids’ aspirations.” (story #1, male respondent).*

*“Rebecca understood – "thanks" to covid – that a healthy work-life balance makes her happier. She will work on herself to become a more balanced person. She will learn how to border the impassable boundaries between work and family. She will learn for herself and her collaborators that if people are happier outside their company, they are also happier inside the company and work better, being more productive.” (story #32, female respondent).*

This first storyline draws a solution enacted by executives to cope with the tension triggered by the juncture between personal and professional domains. In a context where family and work are seen as incompatible and conflicting, especially for executives, the narrators reframe opposing self-definitions of being good executives and involved parents as synergic rather than contrasting. They do this by leveraging new representations of their characters—and themselves—as good executives.

### 4.2 Storyline 2. Withdrawing from the past self: becoming a family man

The second storyline depicts the pandemic as a serendipitous moment that led the good executives to reject their past self-definitions as such to fully embrace the alternative pathways drawn forward. This storyline was sketched by three respondents, two males and one female, and, interestingly, only revolved around the male character, Marc. The pattern begins with Marc finally experiencing high quality time with his family. This experience triggers regrets and a reconsideration of past choices, as Marc imagines how his life could have been different if he had not complied with the occupational schema.

*“Marc, who is at his second marriage after a painful separation from his ex-wife, thought: what if, in my precedent marriage, I would have had this opportunity to stay more with my partner and my family? Would I really have divorced?” (story #10, male respondent).*

While undoing the past and considering alternatives, the executive started revising its priorities rejecting the ideal of work as a moral act. He accepted the unsettling consideration that the sacrifice made to live up work devotion has been delusional.

*“But in 2020, he realized how much he’s missed on, distracted every day by his job. Many people delude themselves with thinking that a company is like a family, and their co-workers are people that they should care about as if they were brothers and sisters. “Bullshit” Marc realizes, “only a family is like a family” (story #3, male respondent).*

In this story, the identity framed around work devotion is openly contested, and the underlying assumption of work as a moral act is rejected. However, this creates incompatibility between the representation of Marc as a good executive and his alternative self. To solve this, Marc rejects being defined by the role, changing his self-definition: from the good executive devoted to the job to an involved husband and father.

*“He quits from the new job and for the first time in his life is unemployed and of course very much worried about it. but he invests some money going back to study and every day he feels better. He also starts engaging with local communities and using his skills for the benefit of other. Eventually he starts working again but part time and finds more fulfilment in giving.” (story #2, female respondent).*

As in the previous storyline, the pandemic enabled Marc to experience an alternative pathway. However, the actualization of the alternative self generates an intense emotional state of regret that prompts Marc to fully withdraw from his past self-definitions. He embraces an alternative view on the self that mirrors that of the narrator: no longer the 24/7-available executive but a present husband and father.

### 4.3 Storyline 3. Withdrawing from the alternative self: reasserting the ideal worker

Finally, four respondents, one male and three females, told a different story, wherein the actualization of the alternative self-prompts Marc and Rebecca to reject the alternative counterfactuals of being involved parents. The main character experienced tension combining opposing self-representations of work and family devotion. Rebecca, for instance, felt the pressure to comply with the societal expectations around being both a good woman and a good executive. She internalized that, to be a good woman, she must invest time in domestic duties. The removal of the scaffolding between work and family has made it difficult for her to demarcate these two domains, creating the pressure to prioritize the former. Rebecca was thus unable to reconcile the competing expectations of being a caring mother and a good executive.

*“Unfortunately, despite her job commitment, Rebecca has to combine home related duties with her regular job resulting in a very stressful period with which she is struggling. Although her husband is supportive and help her in daily chores, Rebecca feels that she’s not being a good mum/wife if she does not take care of crucial aspects of her home.. so she’s doubling up.” (story #7, male respondent).*

Similarly, Marc must deal with availability expectations from family that make it difficult for him to devote himself to work.

*“They started to throw tantrums for the silliest things and Marc could hear them scream from his "home office" aka his bedroom. They started to interrupt important meetings, requiring his attention. Marc had to learn how to play ball with the kids while at the same time running meetings with his team.” (story #17, female respondent).*

Although our sample is too small to draw general conclusions, it may not be coincidental that this storyline predominantly appears among women. More than their male counterparts, female respondents drew on cultural assumptions to infer the incompatibility between the self-representations as good executives and involved parents. Their stories conclude with their main characters—who, by instruction, reflects the narrators’ personal experiences, attitudes, and beliefs—ultimately withdrawing from the alternative self.

*“He started to realize he hated his job, but he felt stuck. He could not do anything about it. He had a family to provide for. To even entertain the idea of resigning and look for another job was crazy. Impossible. It would be irresponsible. You don't quit a good, well paid, job in this economy. No way No how. Nope. Not possible.” (story #17, female respondent).*

*“Looking back, [Marc] considers the choice of completely devoting yourself to work as the only option to build an important career, and a part of him would like to go back to the office as soon as possible, to avoid losing the prestigious position he has at the company” (story #27, female respondent).*

Once the pandemic is over, the main character goes back to the old routine. However, the problematization enacted by the narrators suggests an ongoing reflection in their minds that might influence their professional lives and be self-redefining.

## 5 Discussion

In this study, we examined whether and how executives in masculine contexts developed alternative self-definitions following the alteration of their work-life routine caused by the COVID-19 pandemic, highlighting the role that cultural norms play in this process. Drawing upon the stories told by 32 executives and centered around fictional characters, Marc and Rebecca, we discovered that the sudden shift in work-life habits caused by the pandemic has prompted the emergence of alternative selves, following three distinct patterns or storylines. Initially represented as ideal workers, Marc and Rebecca faced the disruption of the boundaries between work and family domains, which forced them to experience being involved parents, one of their self-definitions if they had made different life choices. The pandemic contributed to make an alternative reality exceptionally salient in the executives’ minds by temporarily actualizing it. This temporary actualization triggered a self-comparison between aspects that previously defined Marc and Rebecca as good executives (e.g., being workaholics) and their actualized, alternative counterfactuals (e.g., being involved parents). Since storytelling depends on the narrators’ ability to process knowledge in an interpretative way (Bruner, 1990), these stories provide an evaluative context for our respondents to reflect on questions around identity and cultural meanings of being either a man or a woman in an executive position. The answers to these questions develop as narrators work through the tensions between past self-definitions and counterfactuals. In the first, most recurrent storyline, Marc and Rebecca deal with the tension by redefining what it means to be a good executive, so that self-definitions and counterfactuals are no longer opposite but synergic. In the second storyline, Marc develops a new self-concept by withdrawing from past self-definitions, embracing the alternative self as an involved parent. In the third storyline, Marc and Rebecca struggle to reject the gendered assumptions around work and family devotion and ultimately decide to withdraw from the alternative self—although the fact that the alternative self has been narrated and problematized might indicate it is still self-redefining. These storylines convey three main contributions to research on work-family interface and research on the alternative self.

### 5.1 The pandemic served as a turning point for executives, eliciting the emergence and development of an alternative self

Existing research suggests that the recent COVID-19 pandemic was a significant turning point, prompting people to reinterpret and renegotiate roles, responsibilities, and priorities across work and family domains (Choudhury et al., 2021). Yet, empirical evidence on this point is still limited. Our study found that the pandemic has effectively elicited alternative views about executives’ self and cultural meanings. These alternative views have led executives to reconsider how they see and understand themselves, outlining counterfactuals that are self-redefining. These findings draw interesting insights on how we think of the impact of the pandemic on the management of the work-family interface and the circumstances that may lead to the rise of alternative selves.

The alternative self is usually described as confined to unactualized realities that are “*intensely imaginary since they are part of a parallel reality*” (Obodaru, 2012, p. 35). Yet, what makes these counterfactuals extremely salient in the context of our study is their temporary actualization. The pandemic made these alternative realities more salient in executives’ minds and turned them into lived experiences, enabling executives to momentarily step outside the predefined path of the good executive aligned with work devotion. This consideration sheds new light on the conditions under which alternative selves arise not merely through retrospective reflection on past turning points (cf. Habermas and Bluck, 2000; Obodaru, 2012), but also through temporary shifts in the timeline of self-development.

**Figure 1:**
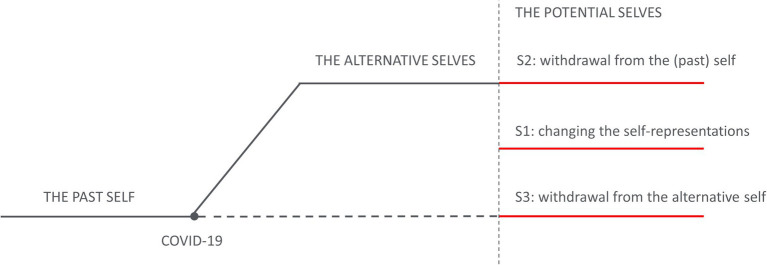
COVID-19 as a nexus of past, present and alternative selves.

Hence, we propose that turning points, as the recent pandemic, can be conceived as *nexuses of past, current, and alternative selves.* This convergence interrupts the relatively stable trajectory of self-development, forcing a detour onto an alternative pathway that might become self-redefining (cf. Figure 1). As the alternative pathway becomes lived experience, executives experienced new tensions between self-definitions as “good executives” and their counterfactuals of “involved parents” that are still perceived as incompatible due to the persistence of prevailing cultural schemas. Dealing with these tensions requires executives to renegotiate who they are and what it means for them to be “good” in these competing roles. This renegotiation, triggered by the actualization of the alternative self, invites a broader reconsideration of the cultural meanings of work and how they shape behaviors and attitudes pertaining to the management of the work-family interface (Williams et al., 2016). Our findings also have implications for how we study alternative selves and turning points, broadening our conceptualization of the alternative self to look beyond purely imaginative realities. Future research should further explore the conditions, other than nexuses, that can trigger self-redefining counterfactuals, possibly leading to the emergence and development of alternative selves.

### 5.2 Story completion as a methodology to study the alternative self and its significance for self-concept development

Researchers have highlighted the opportunities to study the alternative self by using narratives that prompt people to reflect on counterfactuals and progressively internalize them—indeed, we constitute our self-concept through the stories that we tell ourselves and others (Gergen, 2001; Habermas and Bluck, 2000; Obodaru, 2012). Yet, the stories revolving around the descriptions of real events might not capture unactualized realities (Ashforth, 2001; Ibarra and Barbulescu, 2010). Also, they might fall short in grasping those cultural meanings and discourses that enable participants to make sense of their ongoing struggles, desires, and concerns (Clarke et al., 2017). Our study reveals that story completion may offer an approach for retrieving these elements. Our stories revolved around fictional executives who experienced some tensions brought about by the actualization of alternative selves that could have corresponded to narrators’ current self-definitions if they had made different life choices. Although revolving around fictional characters, these narratives were portrayed by actual executives working in masculine contexts, who have been forced to comply with the expectations aligned with the work devotion schema (Cannito and Scavarda, 2020; Chung, 2023; Chung et al., 2021; Chung and Van der Horst, 2020). In other words, these stories are likely to reflect the actual struggles, considerations, tensions, and concerns experienced by respondents, shaping the creation of an alternative self also for the narrators. Indeed, throughout their narratives, all the executives problematized the cultural meanings associated with being an executive with parental duties in a context in which work devotion is still the rule. Such a problematization discloses a layer of self-understanding that goes beyond actual experiences and entails an alternative self (cf. Obodaru, 2012) that can progressively get incorporated into executives’ self-concepts through reflection and narration (cf. Rice and Pasupathi, 2010; Thorne, 2000).

Although future studies are needed to test whether these reflections have eventually led to the actual emergence of new self-concepts for executives, they highlighted dominant themes in executives’ lives and common concerns among respondents, suggesting that they are integrating new experiences, values, and roles that differ from their previously held identities. Most executives in our study rejected traditional self-representations framed around work devotion and gendered roles, leading to a broader understanding of their identity. Therefore, we propose story completion as a methodology for studying the alternative self and its relevance for identity and self-concept development, allowing us to go beyond the limitations of the traditional narrative psychological approach to studying the self (cf. Ashforth, 2001; Ibarra and Barbulescu, 2010).

### 5.3 Rethinking the good executive, considering changes in the construction of occupational scripts

Finally, our findings suggest that the COVID-19 pandemic has triggered a renegotiation of personal preferences, cultural norms, and social identities, affecting how individuals allocate time, energy, and resources across work and family domains. Our stories, indeed, reveal a tendency among executives to reconsider practices and assumptions guided by cultural constructions of their professional responsibilities. This is especially evident, for instance, in the normalization of the use of flexible work policies even in normal conditions, as a tool for juggling between different domains, which arises especially from the first storyline of the executive as a work-life balance advocate. Yet, while the pandemic’s disruption of work-family boundaries may have encouraged more flexible work practices, this alone has not been sufficient to dismantle the cultural foundations of work devotion, which continue to reinforce overwork and gendered role expectations, confirming previous studies (Cannito and Scavarda, 2020; Chung, 2023; de Laat, 2025). Our findings suggest that the work and family devotion schemas still hold significant influence over executives, as evidenced by the identified tensions.

Nonetheless, most respondents in our sample have found ways to make sense of their changing attitudes toward family involvement and their role as executives, revealing an ongoing adaptation process. In the second storyline, Marc finds fulfillment in what could be described as *intensive fatherhood*: a labor-intensive form of parenting where devotion to family overrides other types of commitment. Whereas the first storyline highlights how executives began to craft a new occupational script, where the good executive is no longer defined by blind work devotion, but by the ability to value and apply experiences and skills acquired in domestic life to the workplace (and vice versa). By incorporating a new role as wellbeing advocates into their self-concepts, these executives avoid making either/or choices between family and career, paving the way to a more gender-neutral understanding of work-life balance (cf. Chung et al., 2021).

While we interpret these developments as hopeful signs of an incipient cultural change, they also invite critical reflection. Indeed, these findings echo de Laat (2025)’s concept of “dual devotions,” where both women and men remain highly engaged in childcare and household without sacrificing overwork, because they no longer perceive work and family devotion as competing, mutually exclusive schemas. Although our narratives emphasized balance over full commitment to a single domain, the persistence of work and family schemas suggests that this first storyline may create justifications for intensified expectations across both domains, leading to enhanced exploitation in both, especially for women (Chung, 2023; de Laat, 2025).

Furthermore, the first storyline aligns with the “having it all” ideals that have been problematized, especially in the context of women studies (Seierstad and Kirton, 2015), as women strive to meet unrealistic demands for perfection (McRobbie, 2015). In the COVID-19 aftermath, these ideals seem to have gained heightened significance also for men in high-commitment careers, who increasingly see their involvement with parental duties as part of their personal success. Although our study does not provide evidence of an actual increase in the executives’ work in the household, it highlights a desire for increased involvement with parental duties, especially for men. At the same time, the third storyline suggests that women are still expected to be the primary caregivers, aligning with recent studies (Padavic et al., 2019). The persistence of gendered cultural schemas implies that promoting executives’ involvement with parental duties may further exacerbate tensions between ideals and reality, leading to enhanced frustration that may hinder the necessary cultural change. Therefore, it is critical that organizations recognize the underlying tensions and proactively manage them by supporting men’s roles in households, for example, encouraging them to take parental leaves and promoting care and balance as values for effective management.

## 6 Conclusion

Story completion portrays fictional stories that are imbued with interpretations of characters’ feelings, beliefs, and reconsideration of values. The stories collected and analyzed for this study illustrate how executives rethink themselves while experiencing alternative pathways, and how these thoughts are intertwined with cultural assumptions. However, whether these stories correspond to real changes in executives’ lives at large remains unanswered. Future research should analyze whether these changes in the cultural construction of personal and professional responsibilities have resisted beyond the pandemic, adding a cross-cultural dimension as our sample was composed of executives working in Italy and United Kingdom, both considered as masculine work contexts wherein executives are expected to devote much of their energy to the work with extensive support at home (Dotti Sani, 2014). Further studies should also examine whether the first storyline will remain the most recurrent one, or the third one will become the most prevalent, drawing a way back to old habits and work devotion. Further research is also needed to identify personal, organizational, and societal interventions to sustain the positive changes that some executives experienced during the pandemic. As flexibility becomes a part of the common way of working across companies, what is perceived to be appropriate in an organizational setting might change. This opens the way to renegotiate roles and expectations, paving the way toward a new role model for the good executive focused on community, welfare, and family that should be supported by *ad hoc* organizational policies designed to support this cultural change.

## Data Availability

The raw data supporting the conclusions of this article will be made available by the authors, upon request.
